# College and Hospital Notes

**Published:** 1908-04

**Authors:** 


### COLLEGE AND HOSPITAL NOTES.

---

Professor William Thompson Sedgwick, of the Massachusetts Institute of Technology, will deliver the annual commencement address at the Yale Medical School this year. His subject will be "Preventive Medicine and the Public Health."

Professor Sedgwick was formerly acting president of the Massachusetts Institute of Technology and biologist to the Massachusetts Board of Health. He is curator of the Lowell Institute of Boston.

---

The governors of the New York Skin and Cancer Hospital announce that the following lectures will be given in the Out-Patient Hall of the hospital on Wednesday afternoons, at 4.15 o'clock:

Dr. L. Duncan Bulkley, pathology in its practical bearings upon the treatment of certain diseases of the skin, March 4; also clinical lectures on diseases of the skin, until April 15.

Dr. William Seaman Bainbridge, on the treatment of unremovable cancer, with exhibition of cases, April 22, 1908. The lectures will be free to the medical profession.

